# Tear fluid and complement activation products in tears after ocular surgery

**DOI:** 10.1186/s12886-023-03037-6

**Published:** 2023-07-18

**Authors:** Hiroki Maehara, Koki Norikawa, Keiichiro Tanaka, Yutaka Kato, Akihito Kasai, Tomoko Omori, Takeshi Machida, Hideharu Sekine, Tetsuju Sekiryu

**Affiliations:** 1 Department of Ophthalmology, Fukushima Medical University School of Medicine, Hikarigaoka 1, Fukushima, 960-1247 Japan; 2 Department of Immunology, Fukushima Medical University School of Medicine, Fukushima, Japan

**Keywords:** Tears, Schirmer’s test, Complement activation products, Ocular surface, Tear, Inflammation

## Abstract

**Purpose:**

Due to technological advancements, surgical invasiveness has been reduced. However, cataract surgery has been implicated in causing postoperative inflammation, including dry eye syndrome. The innate immune system may be involved in postoperative inflammation, and complement activation could potentially play a crucial role in defense against pathogens, homeostasis, and wound healing. To investigate changes in the tear film complement activation products (CAPs) and ocular surface after vitrectomy combined with cataract surgery.

**Methods:**

Forty-three patients (23 women; median age, 69 years) were enrolled in this prospective study and underwent phacoemulsification and vitrectomy. We measured Schirmer's test (ST) and CAPs in the tears at baseline (the day before surgery), 4 days and 1 month after the surgery. Tears were collected in microtubes. The CAPs in the tear fluid were analyzed by cytometric bead array.

**Results:**

The median ST (8.5 mm) at baseline increased to 16 mm at 4 days ( *P* < 0.001) and 10 mm at 1 month (*P* = 0.44). The C3a levels (1202 pg/ml) at baseline increased to 2753 pg/ml at 4 days (*P* < 0.001), and 1763 pg/ml at 1 month (*P* = 0.049). The C4a levels (476 pg/ml) at baseline increased to 880 pg/ml at 4 days (*P* < 0.001), and 657 pg/ml at 1 month (*P* = 0.013). The C5a levels (22.6 pg/ml) at baseline increased to 470.9 pg/ml at 4 days (*P* < 0.001), and 38.3 pg/ml at 1 month (*P* = 0.0048). The surgical eyes were divided into the short ST group (≦ 10 mm, *n* = 22) and long ST group (> 10 mm, *n* = 21) based on the preoperative ST values. At 1 month postoperatively, the C3a levels were 2194 pg/ml in the preoperative short ST group and 1391 pg/ml in the long ST group, with significantly higher C3a concentrations in the short ST group (*P* < 0.001).

**Conclusions:**

The CAPs levels in tears increased after vitrectomy combined with cataract surgery. A preoperative deficit in tear secretion might induce prolonged complement activation and delayed recovery of ocular surface parameters postoperatively.

## Introduction

In developed countries, the number of cataract patients is increasing due to the growing elderly population. There is also a rise in the prevalence of dry eye among older individuals[1–3]. Ophthalmologists often perform cataract surgery on patients with dry eye. While advancements in technology have made surgery less invasive, certain factors such as light exposure during surgery, disinfectant toxicity, and evaporation from the ocular surface can contribute to postoperative dry eye [4–8]. This can be problematic for both surgeons and patients, but there is no established treatment for it.

The innate immune system may play a role in postoperative inflammation, and complement, an important component of this system, is involved in protecting against foreign pathogens, maintaining homeostasis, and promoting wound healing [9–11]. Complement activation occurs through three pathways: the classical, lectin, and alternative pathways. Complement is associated with various acute inflammatory processes and helps in defending the host against infections [9–11]. The classical pathway of complement activation is initiated by IgM and IgG immunoglobulins. This leads to the cleavage of C4 and C2, resulting in the activation of C3 and C5. Additionally, the classical pathway can also be activated through the Lectin pathway without the involvement of immunoglobulins. The alternative pathway, on the other hand, operates independently of antibodies and can rapidly activate complement even on the ocular surface. When tissue is damaged, it contains proteases that promote the activation of C3 and C5 [10]. C3 is then cleaved to generate C3a and C3b, while C5 is activated to form C5a and C5b. Ultimately, the complement pathway forms a membrane attack complex (MAC), which leads to cell lysis and death [11]. C3a is known to act as a mediator in inflammatory reactions, affecting polymorphonuclear leukocytes, vasoconstriction, and causing leakage. It is responsible for increased vascular permeability [12]. C5a has been found to mobilize neutrophils and activate basophils, monocytes, and neutrophils, resulting in increased vascular permeability [12, 13]. In ophthalmology, the complement system is involved in age-related macular degeneration [14–18], bacterial keratitis [19], and allergic conjunctivitis [20]. In Sjögren's syndrome and dry eye, tear fluid secretion is decreased, causing inflammation of the ocular surface, which has been reported to be associated with complement activation products (CAPs) [21, 22].

In wound healing, hemostasis, debris clearance, and tissue reconstruction are necessary. At the site of wound healing, platelet aggregate at the wound to establish hemostasis. Degranulation of platelets then induces C3 [12], which generates C3a and C5a that cause migration, activation, and aggregation of mast cells, polymorphonuclear cells, and macrophages [23, 24], all involved in tissue clearance. This cellular response after complement activation may be associated with the wound-healing process in intraocular surgery. Investigating the relationship between the complement activated proteins and ocular surface parameters postoperatively may provide clues to inflammation. We hypothesized that postoperative inflammation would increase in patients who underwent combined vitrectomy and cataract surgery. Therefore, in this study, we examined tear secretion and analyzed the changes in levels of C3a, C4a, and C5a in tears of these patients following the surgical procedures.

### Patients and methods

The study protocol (ID: 30,046) was approved by the Institutional Review Board of Fukushima Medical University, Fukushima, Japan. The research was conducted in accordance with the principles of the Declaration of Helsinki. Written informed consent was obtained from all patients after providing them with a detailed explanation of the study procedures and potential consequences of participation. The study included consecutive patients who underwent combined vitrectomy and cataract surgery for epiretinal membranes and macular holes at Fukushima Medical University Hospital. No patients had received any treatments for dry eye prior to the study. The surgeries were performed by 2 experienced surgeons (YK and AK). Preoperative use of dry eye treatments was not reported among the patients.

The exclusion criteria consisted of a history of previous eye surgeries, the presence of severe conjunctival chalasis, superior limbic keratoconjunctivitis, lid-wiper epitheliopathy, or pterygium. Other exclusion criteria involved systemic diseases that could potentially impact the condition of the ocular surface, such as type 2 diabetes, rheumatoid arthritis, and Sjögren's syndrome.

### Vitrectomy combined with cataract surgery

Levofloxacin 1.5% eye drops (Pfizer, New York, NY, USA) were applied 4 times daily for 3 days preoperatively in all cases. Immediately before surgery, 0.25% polyvinyl alcohol iodine (Rohto Nitten, Nagoya, Japan) was applied, and the surgical field was disinfected according to the package insert.

Vitrectomy combined with cataract surgery was performed using the Constellation Vision System (Alcon Japan Pharma, Tokyo, Japan). Cataract surgery was performed through a 2.4-mm corneal incision, and an intraocular lens was inserted. A 25-gauge pars plana vitrectomy was performed under a non-contact wide-viewing system (Resight Fundus Imaging System; Carl Zeiss Meditec Japan Co., Ltd, Tokyo, Japan). During the surgery, saline was applied to the surgical field to keep the ocular surface hydrated. Postoperatively, the puls planner wound was sutured with 8–0 Vicryl (Mani, Tochigi, Japan) in all cases.

Postoperative eye drops were instilled, i.e., levofloxacin 1.5% eye drop for 2 weeks, 0.1% fluorometholone (Rohto Nitten) for 4 weeks, and bromfenac ophthalmic solution 0.1% (Nisshin, Yamagata, Japan) for 12 weeks postoperatively in all cases.

### The Schirmer's test and tear fluid collection and measurements

The Schirmer's test (ST) on the day before surgery (baseline) and 4 days and 1 month postoperatively to investigate lacrimal fluid secretion. Tear fluid collection was carried out using a non-invasive method during the evaluation of the ocular surface, following established procedures mentioned in previous reports [25]. The same investigator (HM) collected the tear fluid in all cases. To avoid the effect of eye drops on the ocular surface measurements, tear collection was performed at least 1 h after the eye drops were instilled. The patient reclined on a bed in a supine position, while the clinician utilized a micropipette (Drummond Scientific, Broomall, PA, USA) to collect 2 μl of tear fluid from the outer edges of the eyelids. The collected tear fluid was immediately stored at -80° C until analysis. The collected tears were measured using the BD CBA Human Anaphylatoxin Kit (BD Biosciences, San Jose, CA, USA) for CAPs (C3a, C4a, and C5a), according to the manufacturer's instructions [17–19].

### Statistical analysis

The Steel–Dwass tests were used to compare preoperative measurements to those during the follow-up period. Correlation coefficients were performed to identify the association of changes in ST. *P* < 0.05 was considered statistically significant. Statistical analyses were performed using JMP16 software (SAS Institute, Cary, NC, USA).

## Results

A total of 43 patients (23 women, 20 men) with a median age of 69 years (interquartile range [IQR]: 64–73 years) underwent combined vitrectomy and cataract surgery in 43 eyes. The surgical procedure was performed on 1 eye per patient. Among the eyes, 30 had an epiretinal membrane, while the remaining 13 had a macular hole. The median duration of the surgical procedure was 60.0 min (IQR: 49.8–79 min). Table 1 summarizes the clinical data during the follow-up period. All parameters were measured at baseline, 4 days, and 1 month postoperatively, in the surgical eyes.

**Table 1:** Changes of clinical dates during follow-up period

		*P* value vs baseline	*P* value vs 4 days
**base line [Median (IQR)]**			
ST (mm)	8.5 (4–15)	-	-
C3a (pg/ml)	1202 (860–2304)	-	-
C4a (pg/ml)	476 (196–742)	-	-
C5a (pg/ml)	22.6 (15.6–36.0)	-	-
**4 days [Median (IQR)]**			
ST (mm)	16 (7.5–20)	< 0.001	-
C3a (pg/ml)	2753 (1710–4453)	< 0.001	-
C4a (pg/ml)	880 (479–1464)	< 0.001	-
C5a (pg/ml)	470.9 (98.4–1061.4)	< 0.001	-
**1 month [Median (IQR)]**			
ST (mm)	10 (5–15)	0.44	< 0.001
C3a (pg/ml)	1763 (1234–2464)	0.049	< 0.001
C4a (pg/ml)	657 (295–974)	0.013	0.0011
C5a (pg/ml)	38.3 (18.0–66.8)	0.0048	< 0.001

*IQR* interquartile range, *ST* schirmer's test

The ST (mm) values at baseline, 4 days, and 1 month postoperatively were 8.5 (IQR, 4–15), 16 (IQR, 7.5–20), and 10 (IQR, 5–15), respectively, in the surgical eyes. The ST values increased 4 days after the surgery compared to baseline (*P* < 0.001) and decreased 1 month postoperatively compared to baseline (*P* = 0.44).

The C3a levels at baseline, 4 days, and 1 month postoperatively were 1202 pg/ml (IQR, 860–2304), 2753 pg/ml (IQR, 1710–4453), 1763 pg/ml (IQR, 1234–2464), respectively, in the surgical eyes. The C3a levels increased 4 days postoperatively and decreased at 1 month. The differences in the C3a levels between baseline and 4 days and between 4 days and 1 month postoperatively was significant (*P* < 0.001, *P* = 0.049, respectively) (Table 1).

The C4a levels at baseline, 4 days, and 1 month postoperatively were, respectively, 476 pg/ml (IQR, 196–742), 880 pg/ml (IQR, 479–1,464), and 657 pg/ml (IQR, 295–974) in the surgical eyes. The C4a levels increased significantly at 4 days (*P* < 0.001) and 1 month postoperatively (*P* = 0.013) compared to baseline (Table 1).

The C5a level at baseline, 4 days, and 1 month postoperatively were, respectively, 22.6 pg/ml (IQR, 15.6–36.0), 470.9 pg/ml (IQR, 98.4–1,061.4), and 38.3 pg/ml (IQR, 18.0–66.8) in the surgical eyes. The C5a levels increased at 4 days (*P* < 0.001) and 1 month (*P* = 0.0048) postoperatively (Table 1).

**Fig. 1:**
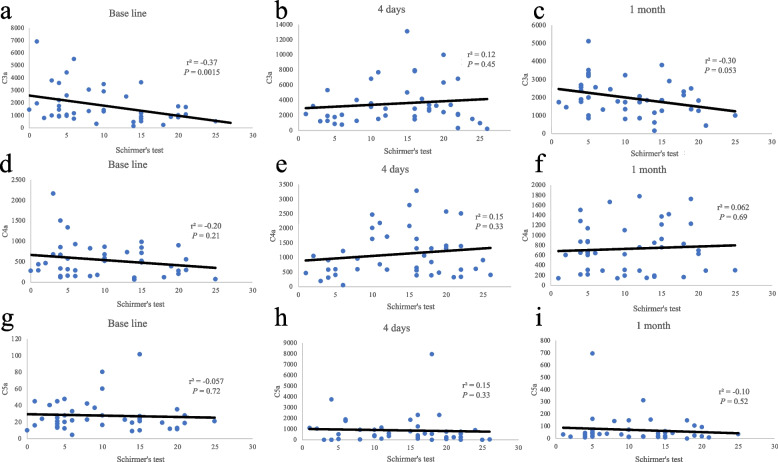
The correartion schirmer’s test and complement activation products. The correlation between Schirmer’s test (ST) and complement activation products (CAPs). There is statistically different between C3a and Schilmer’s test at base line (a). The others had no difference between ST and CAPs (b—i)

The correlation between ST and CAPs in tear fluid was examined (Fig. 1). This figure demonstrated a negative correlation between C3a levels and baseline ST values (*P* = 0.015).

To further investigate the relationship between C3a and ST, we conducted an additional analysis. The surgical eyes were divided into the short ST group (≦ 10 mm, n = 22) and long ST group (> 10 mm, *n* = 21) based on the preoperative ST values. A significant difference was seen in the C3a level between the short and long ST groups (short ST, long ST, 1602 pg/ml [IQR, 960–3193], 882 pg/ml [IQR, 624–1677]) (*P* = 0.041). In the short ST group, the C3a level increased (2210 pg/ml; [IQR, 1506–4328]) at 4 days and remained high (2194 pg/ml; [IQR, 1348–3192]) at 1 month, while in the long ST group, the elevated C3a level 2063 pg/ml; [IQR, 1986–5775] at 4 days decreased to 1391 pg/ml; [IQR, 881–1872] at 1 month (*P* = 0.032) (Table 2). The levels of C4a and C5a in both the short and long ST groups showed an increase at 4 days after surgery and then decreased to the preoperative levels after 1 month (Table 2).

**Table 2:** Relationship between complement activation products and ST during follow-up period

Median(IQR)		Short ST group	*P* value vs baseline	*P* value vs 4 days	Long ST group	*P* value vs baseline	*P* value vs 4 days	*P* value of short ST vs Long ST
C3a (pg/ml)	base line	1602 (960–3193)	-	-	887 (624–1677)	-	-	< 0.001
	4 days	2210 (1506–4328)	0.012	-	3063 (1986–5775)	< 0.001	-	0.29
	1 month	2194 (1348–3192)	0.059	0.076	1391 (881–1872)	0.11	< 0.001	< 0.001
C4a (pg/ml)	base line	386 (171–831)	-	-	519 (241–723)	-	-	0.97
	4 days	762 (451–2061)	< 0.001	-	911 (534–1360)	< 0.001	-	0.95
	1 month	453 (275–876)	0.46	0.0025	620 (295–1030)	0.12	< 0.001	0.60
C5a (pg/ml)	base line	23.4 (16.4–40.4)	-	-	21.2 (11.7–27.6)	-	-	0.42
	4 days	470.9 (93.8–1153.3)	< 0.001	-	565.7 (155.3–670.9)	< 0.001	-	0.70
	1 month	37.0 (16.8–55.3)	0.043	< 0.001	45.6 (22.2–128.1)	0.056	< 0.001	0.53

*ST* schirmer's test, *IQR* interquartile range

## Discussion

In our study, we measured the levels of CAPs in tears and the ST after performing vitrectomy with cataract surgery. Four days after the surgery, we observed a significant increase in ST compared to the baseline measurement. Additionally, the levels of C3a, C4a, and C5a also showed an increase at the 4-day postoperative mark compared to the baseline levels. These elevated levels of CAPs remained high even at the 1-month follow-up.The development of inflammation after cataract surgery is a significant concern for surgeons in this field. Experts have discussed this issue extensively, but there is currently no established protocol for effectively managing postoperative inflammation in patients. Additionally, the innate immune system plays a crucial role in the process of wound healing [26]. However, excessive activation of the complement system can lead to undesirable reactions [27, 28]. Previous experimental studies have indicated a potential association between complement activity and unfavorable postoperative responses [28, 29]. Based on this, we hypothesized that ocular surface parameters might be linked to complement activity during the postoperative period.

The increase in C3a, C4a, and C5a levels 4 days after surgery were considered a common tissue response to surgical injury [30]. The preoperative C3a level in tear fluid showed a negative correlation with the ST value. The levels of C3a and C5a remained elevated immediately after surgery and persisted for 1 month in the short ST group. This suggests that postoperative complement activation through the alternative pathway may be prolonged in eyes with low basal tear fluid secretion. Recent proteome analyses have also indicated the involvement of the complement system in dry eye [31, 32].

This could be associated with reduced CAPs and tear production. In the short ST group examined in this study, the levels of C3a in tear fluid had not returned to their preoperative values even after 1 month of surgery. This indicates that a short ST may contribute to postoperative inflammation. Applying artificial tears to eyes with a short ST could potentially decrease persistent complement activation and alleviate ocular symptoms associated with tear film instability. Measuring ST can help predict postoperative inflammation, and if a short ST is detected, appropriate anti-inflammatory measures and tear fluid replacement may be beneficial. This study is also the first to measure complement activation products in tear fluid. We believe that this research will serve as a foundation for future investigations into inflammation in tear fluid.

### Limitations

The sample size used in the study was too small to establish a clear relationship between dry eye and the surgical impact after the operation. Additionally, we employed absorbable sutures to close the vitrectomy wound as a preventive measure against postoperative infection. However, it is important to note that the biodegradable products (specifically glycol ether) from these sutures can potentially be toxic to the cornea and conjunctiva, leading to inflammation. It is worth mentioning that the sutures were fully absorbed within 2 weeks in all cases, indicating that the impact of the biodegradable materials might be minimal after 1 month. An increase in tear volume after surgery may lead to dilution of proteins in the tear fluid, potentially impacting individuals with pre-existing dry eye conditions. Finally, we did not investigate the relation between the complement system and inflammatory cytokines and cellular immunity. Further studies are needed to determine the role of innate immunity in dry eye.

## Conclusion

Our findings indicate that there was an increase in CAPs after surgery. A reduction in tear secretion before the surgery can lead to prolonged complement activation and a slower recovery of ocular surface parameters following combined vitrectomy and cataract surgery.

## Data Availability

Date are available from Hiroki Maehara (hmaehara@fmu.ac.jp) for researchers who meet the criteria for accessto confidential data.

## Abbreviation

CAPs: complement activation products
ST: Schirmer's test
